# Erecta dislocation of the shoulder joint—A rare injury: About four cases

**DOI:** 10.1002/ccr3.2221

**Published:** 2019-05-26

**Authors:** Malick Diallo, Amadou N. Kassé, Sid’Ahmed Mohamed Limam, Jean C. Sané, Badara Dembélé, Mouhamadou H. Sy

**Affiliations:** ^1^ CHU Souro Sanou Bobo‐Dioulasso Burkina Faso; ^2^ Hopital General de Grand‐Yoff Dakar Senegal; ^3^ Cheikh Zayed Hospital Nouakchott Mauritania; ^4^ CHU Aristide le Dantec Dakar Senegal

**Keywords:** axillary nerve injury, erecta, inferior dislocation, shoulder

## Abstract

A nonresolving axillary nerve injury is a rare associated complication to an inferior dislocation of the shoulder joint. This worsen the midterm outcome of the shoulder. So, neurovascular status must be checked by regular clinical testing and by an electromyography in all cases of inferior dislocation of the shoulder joint.

## 1 INTRODUCTION

The traumatic inferior shoulder dislocation is an unusual injury. The fixed abducted arm above the head so called "hand‐up presentation" gave the name “luxatio humeri erecta” to this dislocation. Inferior dislocations of shoulder joint are sorted in two types: the subglenoid dislocation and the true erecta dislocation.

Four cases of inferior shoulder dislocations among 477 traumatic shoulder dislocations were received in our institution in 9 years. The aim of our study was to discuss their etiological (mechanism and anatomopathology), diagnostic, therapeutic, and follow‐up features.

## 2 CASES

### 2.1 Case 1

**Figure 1:**
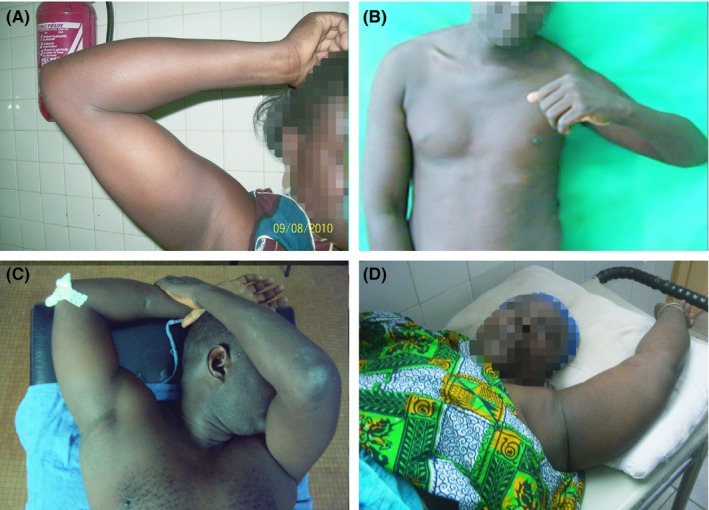
Photographs showing the posttraumatic condition (abducted arms)

**Figure 2:**
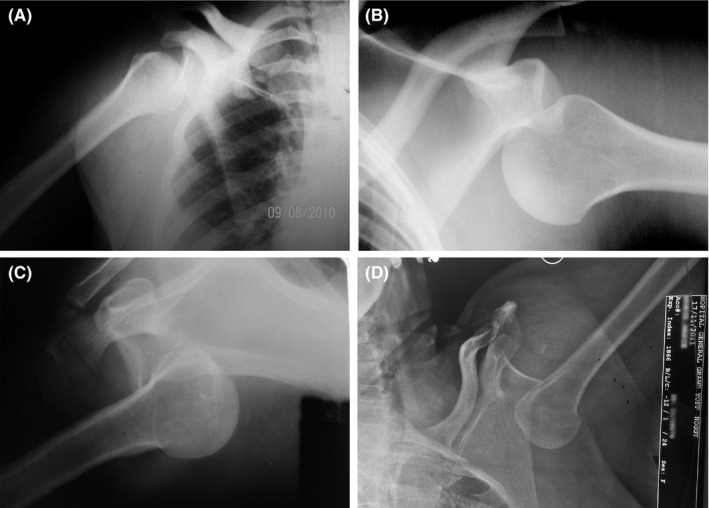
Anteroposterior (AP) view radiographs of shoulders demonstrated shoulder inferior dislocations

A 36‐year‐old woman, right‐handed, sustained a right shoulder trauma after a fall from stairs. She presented to our emergency department with the right arm fixed above the head. At physical examination, it was not associated with vascular and neurologic injury (Figure 1A). The shoulder radiographs showed a subglenoid inferior dislocation of the shoulder joint (Figure 2A). Under general anesthesia, a closed reduction was performed by axial traction. After reduction, the distal pulses and neurological status were normal. The arm was maintained in a sling for 3 weeks. The outcome at 10 months showed a painless shoulder with a full range of motion (Table 1).

**Table 1:** Review of the characteristics of the reported cases

	Age	Sex	Side	MOI	Diagnosis	Complications	Treatment	Follow‐up time (mo)	Outcomes
Case 1	36	F	R	Fall from stairs	Subglenoid	No	CR: Traction	10	Full ROM
Case 2	38	M	L	Fall from a moving bus	Subglenoid	No	CR: Traction		Lost of view
Case 3	42	M	R	Fall from height (4 m)	Erecta	Axillar nerve injury, calcaneus fracture, open‐book pelvic fracture	CR: Two‐step manoeuver	24	Persistant axillar palsy
Case 4	57	F	L	Slipped at house	Erecta	No	CR: Traction	12	Full ROM

Abbreviations: CR, closed reduction method; F, female; L, left; M, male; MOI, mechanism of injury; R, right; ROM, range of motion.

### 2.2 Case 2

A 38‐year‐old man fell down on his left shoulder from a moving bus. The physical presentation (Figure 1B) and the shoulder radiographs (Figure 2B) showed a subglenoid inferior dislocation of the shoulder joint without any associated complication. The shoulder was reduced by an axial traction under sedation. The patient left the hospital and never came back.

### 2.3 Case 3

A 42‐year‐old right‐handed man, driver in public administration, felt down from his four‐meter roof. He sustained a pelvic trauma, a right shoulder trauma, and a right ankle trauma. Physical examination revealed a horizontal unstable pelvic, a fixed right arm above the head, an elbow wound, and a pain swelling right heel (Figure 1C). The distal pulses were palpable but it was a motricity and a sensibility deficit in the axillary nerve territory. Radiographs revealed a Young‐Burgess anteroposterior type 2 (APC‐2) pelvic disruption, a right calcaneal shear fracture, and an erecta inferior dislocation of the right shoulder joint (Figure 2C). A closed reduction was undertaken by the Nho^1^ two‐step manoeuver without anesthesia. Firstly, the humeral head was driven anterior, and then, this secondary anterior dislocation was reduced by Kocher manoeuver. The shoulder was maintained in a sling for 3 weeks. After 24 months, the shoulder joint remained stable with no other dislocation occurrence but still suffering a persistent palsy of axillary nerve. The electromyography (EMG) confirmed a neurotmesis. Pelvic and calcaneal lesions were treated conservatively with a normal gait at 4 months.

### 2.4 Case 4

A 57‐year‐old housewife slipped down on abducted left arm. She complained a severe pain on her left shoulder and the upper limb lied at 180° of abduction (Figure 1D). No neurovascular complication was associated. Radiological views showed an erecta inferior displacement of the humeral head (Figure 2D). A closed reduction was achieved by axial traction. A simple sling was used during 3 weeks for immobilization. She recovered her shoulder with full range of motion after 12 months.

## 3 DISCUSSION

Post‐ traumatic inferior dislocations of the shoulder joint are exceptional.^1^, ^2^, ^3^, ^4^, ^5^, ^6^, ^7^, ^8^, ^9^ Only sporadic cases were described since Middeldorp and Scharm described a case in 1859.^2^

### 3.1 Mechanism of injury

Two main mechanisms of injury (MOI) were described by Davids and Talbott^3^:

1. The hyper‐abduction mechanism, in which the acromion process acts as lever on proximal humerus.
2. The compression mechanism on abducted arm, in which a direct load of the humeral head breaks the capsule.

We think that the hyper‐abduction was the MOI of our cases.

### 3.2 Anatomopathology

Gagey cadaveric studies^10^ showed some similarities in anatomic lesions between anterior and inferior shoulder dislocation: an inferior glenohumeral ligament tear is usual in anterior shoulder dislocations; this tear is longitudinal in inferior dislocations. These explain how an inferior dislocation can be turned in anterior dislocation during attempts of closed reduction.^1^, ^4^

### 3.3 Clinical presentation and complications

The characteristic of inferior dislocations of the shoulder joint is a fixed abducted arm.^1^, ^4^, ^5^, ^10^ Rare cases of inferior dislocations without abducted arm were reported, especially in childhood.^11^ The abducted arm was due to the herniated humeral head through the longitudinal tear of the inferior glenohumeral ligament.^10^ Our cases showed that an abduction around 90° (cases 1 and 2) is related to a subglenoid dislocation and to a true erecta dislocation when the abduction is over 90° (cases 3 and 4). Radiograph views of the shoulder show a displaced humeral head under the glenoid cavity (subglenoid dislocation) and an inverted humerus parallel to the lateral border of the scapula (true erecta dislocation). Axillary nerve palsy was the most reported complication.^6^, ^7^ The nerve position around the humeral neck exposes it to tears when the arm is hyper‐abducted. In our case 2 patient, the axillary nerve sustained a disruption confirmed by the EMG. Others reported complications were an axillary artery injury,^8^ a trochiter fracture,^9^ and a rotator cuff tears.^9^

### 3.4 Treatment and outcome

Conservative treatments gave good results in inferior dislocations of the shoulder joint.^1^, ^3^, ^4^, ^5^, ^6^, ^7^ Closed reduction methods included the classic method^12^ and the Nho et al^1^ two‐step manoeuver. With the classic method, an axial traction was performed on abducted arm with counter traction on the chest.^12^ Then, the arm is driven to its normal position as realized in cases 1 and 3. The two‐step manoeuver transforms firstly an inferior dislocation to a real anterior dislocation of the shoulder before reducing it.^1^ Axillary nerve palsy in shoulder inferior dislocations is commonly a neurapraxia lesion, and it recovers in 2 weeks to 3 years.^7^ The outcome is linked to the axillary nerve injury type. So, it is important to check the palsy with EMG regular controls.^7^

## 4 CONCLUSION

The main clinical aspect of inferior dislocations of the shoulder joint is an abducted arm. Neurovascular status must be checked. An EMG control is important to evaluate an axillary nerve injury and to define the expected midtime outcome.

## CONFLICT OF INTEREST

None declared.

## AUTHOR CONTRIBUTION

MD, ANK, and BD: managed cases 1 and 3. SML and JCS: managed case 4. MHS: managed case 2 and reviewed the manuscript.
